# Downregulation of tumstatin expression by overexpression of ornithine decarboxylase

**DOI:** 10.3892/or.2013.2708

**Published:** 2013-08-29

**Authors:** WEI WANG, CHUN-XIAO XU, GUO-SHENG HOU, YOU-GEN CHEN, JIA-XUAN XIN, XIAN-XI LIU

**Affiliations:** 1 Institute of Biochemistry and Molecular Biology, School of Medicine, Shandong University, Jinan, Shandong 250012, P.R. China; 2 Shandong Center for Disease Control and Prevention, Jinan, Shandong 250014, P.R. China; 3 Quangang Pharmaceutical (SD) Co., Ltd., Jinan, Shandong 250014, P.R. China; 4 Minimally Invasive Urology Center, Provincial Hospital Affiliated to Shandong University, Jinan, Shandong 250021, P.R. China; 5 Institute of Medical Molecular Genetics, Department of Biochemistry and Molecular Biology, Bin Zhou Medical University, Lai Shan, Yan Tai, Shandong 264003, P.R. China

**Keywords:** tumstatin, ornithine decarboxylase, angiogenesis, putrescine

## Abstract

Tumor angiogenesis, a pivotal process for cancer growth and metastasis, requires both upregulation of pro-angiogenic molecules and downregulation of anti-angiogenic molecules. Anti-angiogenesis therapy represents a promising way for cancer treatment. Tumstatin, a novel endogenous angiogenesis inhibitor, inhibits endothelial cell proliferation, pathological angiogenesis and tumor growth. Ornithine decarboxylase (ODC), overexpressed in various cancers, is associated with cell transformation, tumor invasion and angiogenesis. We found that the expression of tumstatin was suppressed in ODC-overexpressing human cancer cells and renal carcinoma tissues. We presumed that ODC overexpression may downregulate the expression of tumstatin. To be able to test this hypothesis, we generated HEK293 cells that overexpress ODC (ODC transfectants) and characterized the following experimental groups: PBS-treated group, mock transfectants, ODC transfectants, ODC transfectants transfected with pcDNA-ODCr (an antisense ODC-expressing plasmid) group and putrescine-treated group. The effect of ODC overexpression on tumstatin expression was examined by reverse transcriptase-polymerase chain reaction (RT-PCR), western blot analysis and dual luciferase reporter assay. ODC-overexpressing cells and putrescine-treated cells showed suppressed tumstatin mRNA and protein expression, and decreased tumstatin gene promoter activity. Thus, ODC overexpression suppresses the expression of tumstatin, which may provide fundamental evidence for the combination of anti-angiogenic therapy and conventional therapy for cancer treatment.

## Introduction

Tumstatin has been found to be a target of autoantibodies in patients with Goodpasture syndrome (1). This novel endogenous anti-angiogenic molecule is the bioactive NCI domain (28 kDa) of colIVα3 chain, liberated from the basement membrane through cleavage by matrix metalloproteinase (2–4). Tumstatin can specially inhibit proliferation of endothelial cells, cause G1 arrest of vascular endothelial growth factor (VEGF)- and basic fibroblast growth factor (bFGF)-stimulated endothelial cells, induce apoptosis of proliferating endothelial cells and consequently inhibit pathological angiogenesis (5–7). Tumor growth in many mouse xenograft models treated with tumstatin is suppressed because tumstatin can induce endothelial cell-specific apoptosis (8–10). It is currently considered to be a promising anti-angiogenic and antitumor agent for its unique property of causing ‘tumor stasis’ (11).

Polyamine, comprised of putrescine, spermine and spermidine, not only plays an important role in the maintenance of normal cell function, but also is involved in the formation of multiple malignant phenotypes including tumor angiogenesis (12–14). Ornithine decarboxylase (ODC), the first rate-limiting enzyme of polyamine biosynthesis, catalyzes the decarboxylation of ornithine to produce putrescine (15,16). ODC, associated with cell growth, proliferation, transformation and angiogenesis, has been shown to be overexpressed in various cancers (15,17–19). Nude mice inoculated with ODC-overproducing NIH3T3 cells developed well-vascularized tumors which were vascularized abundantly (18). This tumor neovascularization was elicited not by VEGF and bFGF, but by a novel angiogenesis factor which promotes endothelial cell proliferation and migration. Concomitant to this is the production of thrombospondins, an inhibitor of angiogenesis, which appear to be decreased in ODC-transformed cells. On the other hand, DL-α-difluoromethylornithine (DFMO), an irreversible inhibitor of ODC, can inhibit tumor angiogenesis and subsequent tumor growth (20,21). The inhibitory effect of DFMO on B16 melanoma cells was much less than that on bovine pulmonary artery endothelial cells. Therefore, Takahashi *et al* presumed that the antitumor effect of DFMO is mostly attributed to tumor angiogenesis inhibition by polyamine depletion (20).

Nemoto *et al* found that ODC overexpression facilitates angiogenesis by suppressing the expression of endostatin, which is also an endogenous angiogenesis inhibitor (22). We presumed that overexpression of ODC may downregulate the expression of tumstatin. To overexpress ODC, we generated the plasmid pcDNA-ODC and transfected it into HEK293 cells to establish ODC transfectants. Subsequently, the effect of ODC overexpression on tumstatin expression was examined in the following cell lines: PBS-treated cells, mock transfectants, ODC transfectants, ODC transfectants transfected with pcDNA-ODCr, and putrescine-treated cells. The data presented here show that ODC overexpression downregulates the tumstatin level.

## Materials and methods

### Sample collection

Thirty-eight cancerous samples paired with noncancerous tissues adjacent to the cancer tissue of kidney were obtained from Shandong Provincial Hospital, Shandong, China. Informed consent was obtained from all patients before surgery.

### Plasmid construction

Full-length human ODC gene sequence was amplified using the following primers: 5′-CCGTCTAGA ATGAACAACTTTGGTAATGA-3′ and 5′-AGAAAGCTTCT ACACATTAATACTAGCCG-3′ (enzyme recognition sites are underlined). An ODC-overexpressing plasmid pcDNA-ODC was constructed by inserting the ODC cDNA into an expression vector pcDNA3.1/Myc-His(−)A, and the correct plasmid was identified by restriction enzyme digestion and DNA sequencing. The antisense ODC expressing vector pcDNA-ODCr was constructed as described previously (23).

### Cells, stable transfection and treatment

HEK293 cells (human embryonic kidney cell line), ACHN cells (human renal carcinoma cell line), HELF6 cells (human embryonic lung fibroblast cell line) and A549 cells (human lung carcinoma cell line) were cultured in Dulbecco's modified Eagle's medium (DMEM), DMEM, RPMI-1640 medium and Ham's/F-12 medium, respectively, supplemented with 10% fetal bovine serum, 100 U/ml of penicillin and 100 μg/ml streptomycin.

HEK293 cells transfected with pcDNA3.1/Myc-His(−)A and pcDNA-ODC were selected using G418. At 3–4 weeks later, the positive clone was picked, digested with trypsinase and further cultured to establish mock and ODC transfectants.

Untreated HEK293 cells, mock transfectants and ODC transfectants were plated in 6-well plates. At 24 h later, untreated HEK293 cells were treated with PBS or 100 μM putrescine, while the ODC transfectants were transfected with pcDNA-ODCr. All five groups of cells (PBS-treated, mock transfectants, ODC transfectants, pcDNA-ODC and pcDNA-ODCr transfectants, and putrescine-treated group) were harvested 72 h post-treatment for further analysis.

### Semi-quantitative reverse transcriptase-polymerase chain reaction (RT-PCR)

Total RNA was isolated from kidney tissues and cells were treated as described above and then used for reverse transcription according to the manufacturer's protocol (Fermentas). PCR analysis was performed using the following primers: β-actin (5′-CCACTGGCATCGTGATGGAC-3′ and 5′-GCGGATGTCCACGTCACACT-3′), ODC (5′-CCGTCTAGAATGAACAACTTTGGTAATGA-3′ and 5′-AGAAAGCTTCTACACATTAATACTAGCCG-3′), tumstatin (5′-GACGGTACCATGCCAGGTTTGAAAGGAA-3′ and 5′-GGCTCGAGTCAGTGTCTTTTCTTCATGC-3′), endostatin (5′-ACGCATCTTCTCCTTTGACG-3′ and 5′-TGGCTACTTGGAGGCAGTCA-3′) and VEGF (5′-CATCCCTGTGGGCCTTGCTC-3′ and 5′-GCTCACCGCCTCGGCTTGTC-3′). Band intensities for ODC, tumstatin, endostatin and VEGF fragment were quantified and normalized to the intensity of the β-actin signal.

### Western blot analysis

Western blot analysis was performed using cell lysates. ODC was observed using the specific mouse anti-human monoclonal antibody (Sigma). Tumstatin was detected with the antibody prepared by our laboratory (24). Bands were visualized by the electrochemiluminescence protein detection system (Millipore). An immunoblot with antibody against β-actin was used as a control.

### Dual luciferase reporter assay of tumstatin gene promoter

The tumstatin gene promoter was amplified from total DNA extracted from human peripheral blood, using the following primers: 5′-GGTACCAGCAACATCTGCGATATGGTC-3′ and 5′-AAGCTTTCAGAGCCTGGGCGAGTC-3′. The amplified products were digested, purified and inserted into the pGL3-basic null vector to form the reporter construct pGL-tumstatin (2.2 kb). HEK293 cells were plated in 24-well plates at a density of 1×10^5^ cells/well prior to transfection. Using Lipofectamine™ 2000, the cells were co-transfected with pRL-TK and the following constructs: pGL3-basic (blank control), pGL3-control (positive control) or pGL-tumstatin (2.2 kb). The cells were collected 48 h later for the promoter activity assay.

Using the same procedure, pGL-tumstatin (2.2 kb) and pRL-TK were co-transfected into HEK293 cells treated as described above (categorized into five groups). The cells were harvested 48 h later for the promoter activity assay using the dual-luciferase reporter system (Promega) according to the manufacturer's protocol.

### Statistical analysis

Data were expressed as the means ± SD. Correlation analysis and ANOVA were performed by SPSS 13.0 statistical software package and P<0.05 was considered statistically significant.

## Results

### The expression of ODC and tumstatin in renal tissues and cells

We detected the expression of ODC and tumstatin in various tumor cells. In the ACHN and A549 cells, ODC was overexpressed, while the expression of tumstatin and endostatin were remarkably suppressed in comparison with the corresponding normal cells (HEK293 and HELF6, respectively), as determined by RT-PCR and western blot analysis (Table I).

**Figure 1:**
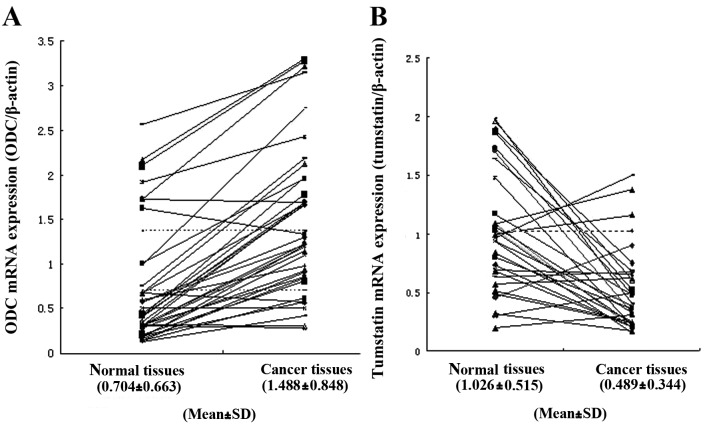
mRNA expression levels of ODC and tumstatin in renal tissues. (A) ODC mRNA expression level measured by RT-PCR. Band intensities were quantified and normalized to the intensity of the β-actin signal. The dashed lines indicate the mean and mean + SD levels. (B) Tumstatin mRNA expression level in ODC-overexpressing renal cancer tissues. The dashed line indicates the mean level.

Subsequently, RT-PCR was performed to detect the expression levels of ODC and tumstatin in human renal cancer and adjacent normal tissues. ODC mRNA overexpression was detected in 32 of 38 cancerous tissues, and not in any of the corresponding normal tissues (Fig. 1A). In these 32 ODC-overexpressing kidney cancerous samples, 24 had downregulated tumstatin mRNA expression when compared with the adjacent normal tissues (Fig. 1B). Statistical analysis revealed a correlation between ODC gene expression and tumstatin expression (P<0.05).

### Establishment of mock transfectants and ODC transfectants

Full-length human ODC cDNA (1,386 bp) was cloned into pcDNA3.1/Myc-His(−)A expression vector to generate the eukaryotic expression plasmid pcDNA-ODC, which was identified and confirmed by restriction enzyme digestion and DNA sequencing. Subsequently, RT-PCR and western blot analysis were performed to detect whether the recombinant plasmid could be expressed in eukaryotic cells (HEK293) (data not shown).

**Figure 2:**
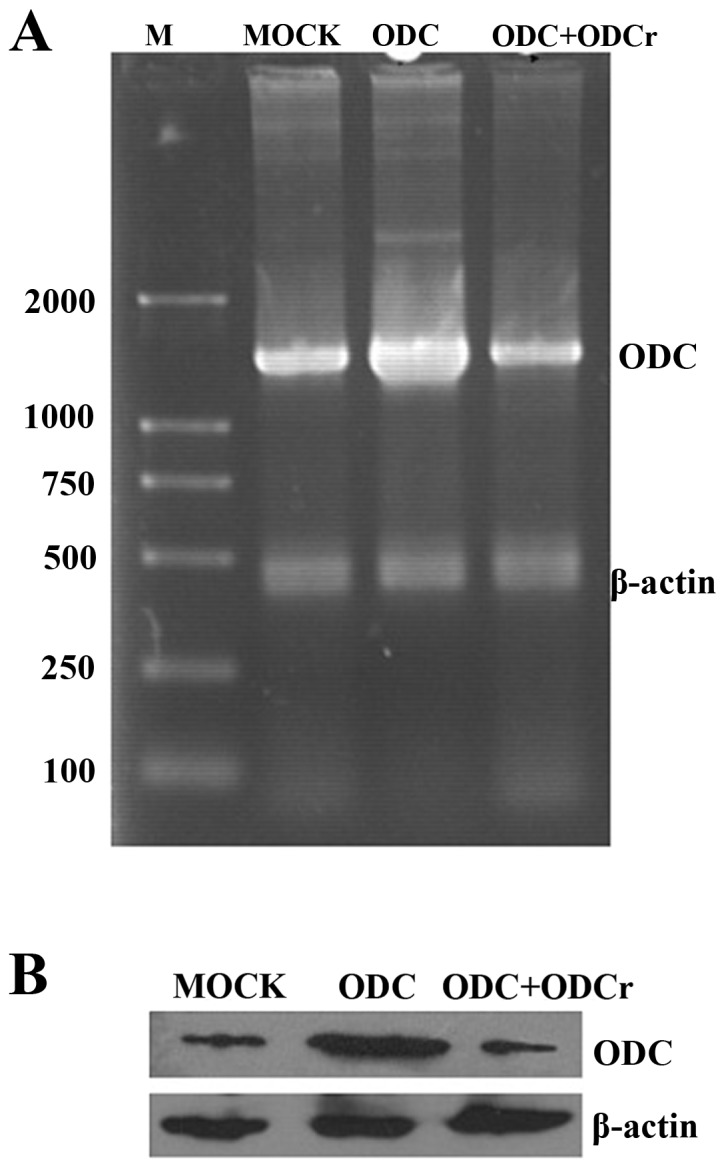
The expression of ODC mRNA and protein in mock transfectants, ODC transfectants and ODC transfectants transfected with pcDNA-ODCr. (A) RT-PCR analysis was performed to detect ODC mRNA expression. Lane M, DL-2000 marker; lane Mock, mock transfectants; lane ODC, ODC transfectants; lane ODC + ODCr, ODC transfectants transfected with pcDNA-ODCr. β-actin was used as an internal control. (B) Western blot analysis with anti-ODC monoclonal antibody. Lane Mock, mock transfectants; lane ODC, ODC transfectants; lane ODC + ODCr, ODC transfectants transfected with pcDNA-ODCr. Equal loading was verified using an anti-β-actin antibody.

HEK293 cells transfected with pcDNA3.1/Myc-His(−)A and pcDNA-ODC were selected in the presence of G418 for 3–4 weeks, and the surviving cells were established as mock transfectants and ODC transfectants, respectively. Compared with mock transfectants, the expression level of ODC mRNA (Fig. 2A) and protein (Fig. 2B) in ODC transfectants was increased by 200 and 196%, respectively. However, the ODC mRNA and protein expression level in ODC transfectants transfected with pcDNA-ODCr recovered to the same level as that in mock transfectants (Fig. 2), which indicates that pcDNA3.1-mediated antisense ODC could inhibit the expression of ODC in ODC tranfectants.

### ODC overexpression suppressed tumstatin expression

**Figure 3:**
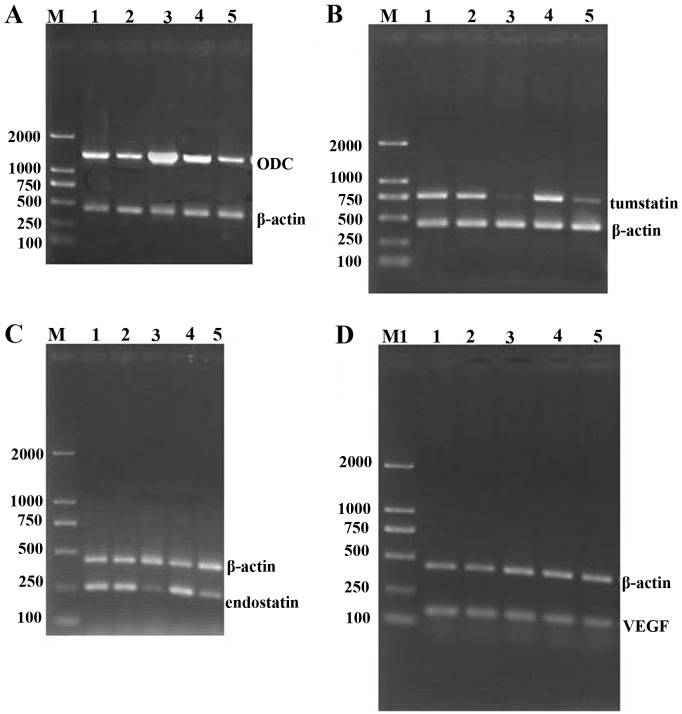
mRNA expression of ODC, tumstatin, endostatin and VEGF in HEK293 cells after treatment. (A) A 1,286 bp band representing the ODC gene, (B) a 750 bp fragment representing the tumstatin gene, (C) a 261 bp fragment representing endostatin and (D) 156 bp fragment representing VEGF were amplified by RT-PCR and visualized. β-actin was also amplified as an internal control. Lane M, DL-2000 marker; lane 1, PBS-treated group; lane 2, mock transfectants; lane 3, ODC transfectants; lane 4, ODC transfectants transfected with pcDNA-ODCr; lane 5, putrescine-treated group.

**Figure 4:**
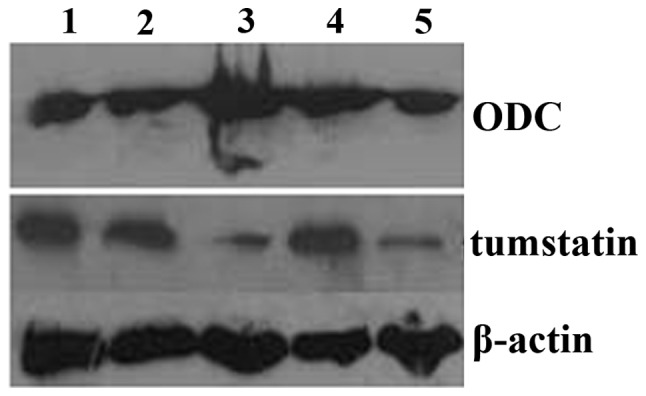
Western blot analysis was performed to detect the expression of ODC and tumstatin gene in cells after treatment. Lane 1, PBS-treated group; lane 2, mock transfectants; lane 3, ODC transfectants; lane 4, ODC transfectants transfected with pcDNA-ODCr; lane 5, putrescine-treated group. β-actin was detected with an anti-β-actin antibody as an internal control.

In order to examine the effect of ODC overexpression on tumstatin expression, we generated transfectants overexpressing ODC and mock transfectants containing vector alone. Then HEK293 cells were subjected to different conditions: PBS treatment, mock transfection, ODC transfection, pcDNA-ODC + pcDNA-ODCr (ODC transfectants transfected with pcDNA-ODCr) and putrescine treatment. The expression levels of ODC and tumstatin in HEK293 cells treated as described above were detected by semi-quantitative RT-PCR and western blot analysis. ODC mRNA (Fig. 3A) and protein (Fig. 4) were overexpressed in ODC transfectants, and the elevated ODC expression level approached that of mock transfectants in the pcDNA-ODC + pcDNA-ODCr group. No change in ODC expression level was detected in the putrescine-treated group, as compared with PBS-treated group (Table II). However, the expression of tumstatin at both the mRNA (Fig. 3B) and protein levels (Fig. 4) was significantly less in ODC transfectants and putrescine-treated group than in PBS-treated group. The suppression of tumstatin expression in ODC transfectants was rescued after transfection of pcDNA-ODCr (Table II). We analyzed the expression of endostatin and VEGF mRNA in treated HEK293 cells. The expression of endostatin in ODC transfectants and putrescine-treated group was significantly inhibited, but the downregulation of endostatin expression in ODC transfectants was restored upon transfection of pcDNA-ODCr (Fig. 3C), while VEGF mRNA expression level remained unchanged (Fig. 3D) (Table II).

**Figure 5:**
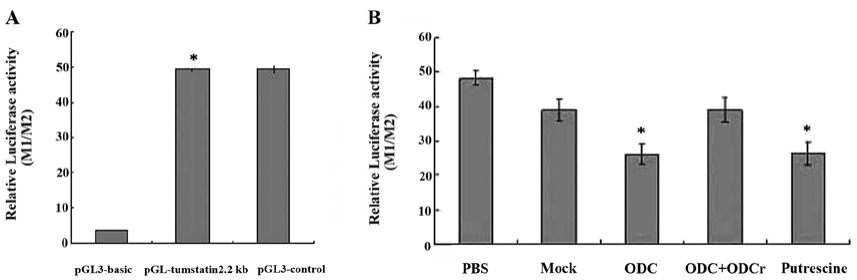
Analysis of the tumstatin promoter activity. (A) Tumstatin gene promoter activity in HEK293 cells. pGL-tumstatin (2.2 kb) and pRL-TK were co-transfected into HEK293 cells to detect whether the amplified fragment possesses promoter activity. pGL3-basic and pGL3-control were used as negative control and positive control, respectively. (B) Dual-luciferase reporter assay was used to detect the effects of the treatment on promoter activity of tumstatin gene. Each bar represents the mean ± SD of three experiments.

After demonstrating that ODC overexpression results in the downregulation of tumstatin mRNA and protein levels, we then examined the effect of ODC overexpression on the tumstatin gene promoter. A luciferase reporter plasmid pGL-tumstatin 2.2 kb containing the full-length promoter region (2,149 bp) was constructed and identified by restriction enzyme digestion and DNA sequencing. Subsequently, the tumstatin gene promoter luciferase reporter plasmid was transfected into HEK293 cells and luciferase activity assay was performed. As shown in Fig. 5A, wherein M1/M2 represents the relative luciferase activity, the 2,149 bp fragment exhibited promoter activity. pGL-tumstatin (2.2 kb) exhibited pronounced promoter activity (49.25±0.46) to the same degree as that of the positive control pGL3-control (49.3±1.2). Cells transfected with pGL3-basic (7.1±0.01) showed no promoter activity.

We next investigated whether pGL-tumstatin (2.2 kb) could be regulated by ODC overexpression. Luciferase activity assay results showed that the activity of pGL-tumstatin (2.2 kb) decreased to 45.8% upon ODC overexpression (26.12±2.8) and to 45.2% upon putrescine addition (26.36±3.42), compared with the PBS-treated group (48.16±2.05). However, the luciferase activity of pGL-tumstatin (2.2 kb) in ODC transfectants matched that in mock transfectants (38.96±3.22) upon transfection with the antisense plasmid pcDNA-ODCr (39.04±3.68) (Fig. 5B). These results indicate that ODC overexpression and putrescine suppressed the expression of tumstatin by inhibiting promoter activity.

## Discussion

Angiogenesis, characterized by generation of new capillaries, plays an important role in physiological processes such as wound healing and in pathological disorders such as cancer (25,26). Tumor angiogenesis is indispensable for solid tumor growth and metastasis (27,28). Neovascularization in tumors supplies tumor with oxygen and nutrition, stimulates tumor progression by paracrine secretion and facilitates the hematogenous metastasis of tumor cells (29). Targeting the blood vessels feeding tumors could result in tumor starvation and tumor regression. Therefore, tumor angiogenesis presents an essential target of therapeutic intervention for cancer.

Tumor angiogenesis requires both upregulation of angiogenic stimulators such as VEGF, bFGF, angiogenin and downregulation of endogenous angiogenic inhibitors such as tumstatin, endostatin and thrombospondin (30). Endogenous angiogenic inhibitors and synthetic inhibitors or antibodies to angiogenic stimulators have been investigated as potential therapeutic agents against tumors because of their promising antitumor activity (27,28).

Tumstatin, a novel endogenous angiogenic inhibitor, specifically suppresses proliferation of endothelial cells, induces apoptosis of endothelial cells, and inhibits pathological angiogenesis and tumor growth (31). It has distinct antitumor properties with an N-terminal (amino acids 54–132) possessing anti-angiogenic activity and a C-terminal (amino acids 185–203) having antitumor cell activity. Tumstatin exerts an antitumor effect by binding to αvβ3 integrin in endothelial cells and melanoma cells (5,7,10). Endostatin, another endogenous inhibitor of angiogenesis, originates from the α1 chain of type XVIII collagen (32). Tumstatin and endostatin share a 14% amino acid homology and exhibit distinct anti-angiogenic activities (6). Anti-angiogenesis effect of tumstatin is ten times more potent than that of endostatin, therefore tumstatin is regarded as a promising anticancer therapeutic candidate (6,33). However, tumstatin alone failed to achieve tumor regression. It might have an adjuvant role in tumor treatment and is effective against tumors when administered in combination with conventional therapy (33). Many studies have demonstrated that the addition of tumstatin or endostatin increases the antitumor efficacy of conventional therapies (34–36). Thus the combined treatment of these agents could be used for targeting cancer in the future.

The role of ODC and polyamine in cancer has been a focus of many research studies. Elevated ODC activity has been detected in many cancers and thought to be associated with cell transformation, tumor invasion and angiogenesis (14,16–19). The ODC gene is considered to be an oncogene because overexpression of ODC results in malignant transformation of NIH3T3 cells (18,37). B16 melanoma-induced angiogenesis, rapid neovascularization and tumor growth are inhibited by DFMO by irreversibly inactivating ODC, and these inhibitions are reversed by exogenous putrescine and spermidine (20,38). Due to this, DFMO has been used as chemotherapeutic agent in clinical trials for cancers, although it exhibits dose-limiting toxicity.

In the present study, we found that ODC-overexpressing human cancer cells (ACHN and A549) and renal cancer tissues have reduced expression of tumstatin. ODC overexpression can downregulate the production of thrombospondin and suppress the gene expression of type XVIII collagen and endostatin (20,22). Therefore, we hypothesized that ODC overexpression can inhibit the expression of tumstatin. To examine the effect of ODC overexpression on tumstatin expression, we generated an ODC overexpressing plasmid pcDNA-ODC and established ODC-overexpressing HEK293 cells.

In this study, cells subjected to five different conditions were examined: PBS treatment (control group), mock transfection, ODC transfection, ODC transfectants transfected with pcDNA-ODCr, and putrescine treatment. The overexpression of ODC in ODC transfectants was suppressed by the antisense plasmid pcDNA-ODCr. This experimental setup allowed us to examine the expression level of tumstatin relative to the level of ODC. The effect of the antisense plasmid pcDNA-ODCr on ODC was similar to that of DFMO. RT-PCR and western blot results showed that ODC overexpression and putrescine inhibited the expression of tumstatin mRNA and protein, while the suppression of tumstatin expression in ODC transfectants was rescued after transfection of pcDNA-ODCr. The expression level of VEGF mRNA remained unchanged, demonstrating that the effect of ODC overexpression and putrescine on promoting angiogenesis was not associated with VEGF. This is consistent with a previous report by Nemoto *et al*(22). In order to better understand the negative effect of ODC on tumstatin expression, we examined the effect of ODC on tumstatin gene promoter activity. The results from the dual luciferase reporter assay indicate that ODC overexpression and putrescine suppressed the expression of tumstatin by inhibiting tumstatin promoter activity. Taken together, these results support that ODC may promote tumor angiogenesis by suppressing tumstatin expression in many cancers. This finding provides novel evidence for the efficacy of combining anti-angiogenic therapy with conventional therapy for cancer treatment.

## Figures and Tables

**Table I:** The expression of ODC, tumstatin and endostatin in cells.

		ODC		Tumstatin		
				
Sample	n	mRNA	Protein	mRNA	Protein	Endostatin mRNA
HEK293	3	0.90±0.05	0.27±0.09	0.91±0.66	0.69±0.23	1.29±0.05
ACHN	3	2.39±0.43^a^	0.93±0.13^a^	0.30±0.02^a^	0.11±0.10^a^	0.50±0.10^a^
HELF6	3	1.25±0.61	0.65±0.14	1.01±0.06	0.52±0.10	1.20±0.16
A549	3	2.93±0.65^a^	1.45±0.06^a^	0.20±0.03^a^	0.07±0.12^a^	0.35±0.26^a^

a P<0.05 vs. the corresponding normal cells.

**Table II:** The expression of ODC, tumstatin, endostatin and VEGF in treated HEK293 cells.

	Treatment				
	
Detection	PBS	Mock transfectants	ODC transfectants	ODC + ODCr	Putrescine
ODC mRNA	1.89±0.02	1.91±0.43	3.31±0.29^a^	1.90±0.21	1.60±0.41
ODC protein	0.237±0.04	0.245±0.03	0.598±0.14^a^	0.308±0.04	0.239±0.01
Tumstatin mRNA	1.13±0.23	1.16±0.37	0.26±0.34^a^	1.16±0.16	0.34±0.32^a^
Tumstatin protein	0.235±0.07	0.259±0.05	0.078±0.04^a^	0.235±0.02	0.114±0.06^a^
Endostatin mRNA	0.97±0.25	0.96±0.09	0.31±0.07^a^	1.08±0.10	0.40±0.24^a^
VEGF mRNA	0.76±0.26	0.75±0.34	0.74±0.38	0.76±0.22	0.75±0.39

a P<0.05 vs. PBS-treated group.
